# Weighted Gene Co-Expression Network Analysis Identifies ANGPTL4 as a Key Regulator in Diabetic Cardiomyopathy *via* FAK/SIRT3/ROS Pathway in Cardiomyocyte

**DOI:** 10.3389/fendo.2021.705154

**Published:** 2021-09-20

**Authors:** Lei Dai, Yang Xie, Wenjun Zhang, Xiaodan Zhong, Mengwen Wang, Hongcheng Jiang, Zhen He, Xiaolei Liu, Hesong Zeng, Hongjie Wang

**Affiliations:** ^1^Division of Cardiology, Department of Internal Medicine, Tongji Hospital, Tongji Medical College, Huazhong University of Science and Technology, Wuhan, China; ^2^Hubei Key Laboratory of Genetics and Molecular Mechanism of Cardiologic Disorders, Huazhong University of Science and Technology, Wuhan, China

**Keywords:** diabetic cardiomyopathy, weighted gene co-expression network analysis (WGCNA), ANGPTL4, focal adhesion kinase, NADPH oxidase, apoptosis, SIRT3

## Abstract

**Background:**

Diabetic cardiomyopathy (DbCM) is characterized by initial impairment of left ventricular relaxation followed by contractile dysfunction. Despite intensive research, the exact mechanism remains so far unsolved.

**Methods:**

We constructed weighted gene co-expression network analysis (WGCNA) to screen gene modules that were closely related with DbCM based on the GSE5606 dataset, which contained expression data of the cardiac left ventricle in a rodent model of streptozotocin (STZ)-induced DbCM. Then, the most related hub gene, angiopoietin-like 4 (ANGPTL4), was selected for functional *ex vivo* and *in vitro* assays. In our experiments, STZ-induced diabetic mice (C57BL/6J) and human cardiomyocytes (AC16) were used to study the functional roles and potential mechanisms of ANGPTL4 in DbCM.

**Results:**

WGCNA analysis revealed the yellow and green modules were most correlated with DbCM, and identified ANGPTL4 as one of the most significantly upregulated hub genes (*ANGPTL4*, *ACOT1*, *DECR1*, *HMGCS2*, and *PDK4*). Consistent with the bioinformatic analysis, the amount of ANGPTL4 was significantly upregulated in diabetic mouse heart. DbCM group, compared with the control group, had increased phosphorylation of focal adhesion kinase (FAK), reduced SIRT3 expression, increased SOD2 acetylation, upregulated NADPH oxidase activation, elevated reactive oxygen species (ROS) produciton, and enhanced apoptosis in the diabetic mouse heart. Moreover, ANGPTL4 induced apoptosis *via* FAK/SIRT3/ROS pathway in human cardiomyocytes (AC16) under high glucose condition *in vitro.*These effects were abrogated by treatment of two independent siRNA for ANGPTL4, whereas exogenous recombinant ANGPLT4 protein treatment exacerbated those effects in AC16.

**Conclusion:**

We found *ANGPTL4*, *ACOT1*, *DECR1*, *HMGCS2*, and *PDK4* were significantly increased in diabetic heart. ANGPTL4 could promote cardiac apoptosis *via* a FAK/SIRT3/ROS dependent signaling pathway in DbCM.

## Introduction

Diabetes mellitus (DM) is one of the fastest growing global health emergencies of the 21^st^ century. In 2019, it is estimated that 463 million people have diabetes, and this number is projected to reach 578 million by 2030 and 700 million by 2045 (1). Cardiovascular complications are the primary cause of mortality and morbidity in diabetic patients (2). Diabetes increases the risk of heart failure approximately two-fold, independent of coronary artery disease and other comorbidities (3). Interestingly, Rubler et al. found that a subset of diabetic patients develops left ventricular dysfunction in the absence of coronary artery disease, hypertension, or vascular disease, and they termed this diabetic cardiomyopathy (DbCM) (4). And further histological studies showed that DbCM is charactherized by diffuse fibrosis, myofibrillar hypertrophy, microvascular disease, and deposition of acid mucopolysaccharide material (4, 5).

Based on the clinical and histopatholigical features of DbCM, many novel mechanisms have been graudually identified, including systemic metabolic disorder, mitochondrial damage, oxidative stress, microcirculation disorder, inflammation, and heart remodeling (5–7). Despite much progress on DbCM research in the past decade, the precise pathogenesis still remains unclear (8). Moreover, the progression of DbCM cannot be effectively reversed or even halted by traditional heart failure therapies or tight blood glucose control (9), making it a major unmet clinical need among cardiovascular diseases.

With the gradual development of microarray and high-throughput sequencing, it is possible to further explore novel biomarkers and therapeutic targets for DbCM. The systems biology algorithm of weighted gene co-expression network analysis (WGCNA) was constructed to find clusters (modules) of highly correlated genes by building a scale-free gene co-expression network, and identify gene modules and hub genes that were highly co-regulated and closely related with clinical traits (10). In this study, we constructed WGCNA by a public microarray dataset and recognized hub genes related to DbCM. The most related hub gene, angiopoietin-like 4 (ANGPTL4), was selected for subsequent functional *ex vivo* and *in vitro* assays. Our findings provided novel clues awaiting further validation for DbCM treatment in future clinical trials.

## Materials and Methods

### Data Collection and Preprocessing

**Figure 1:**
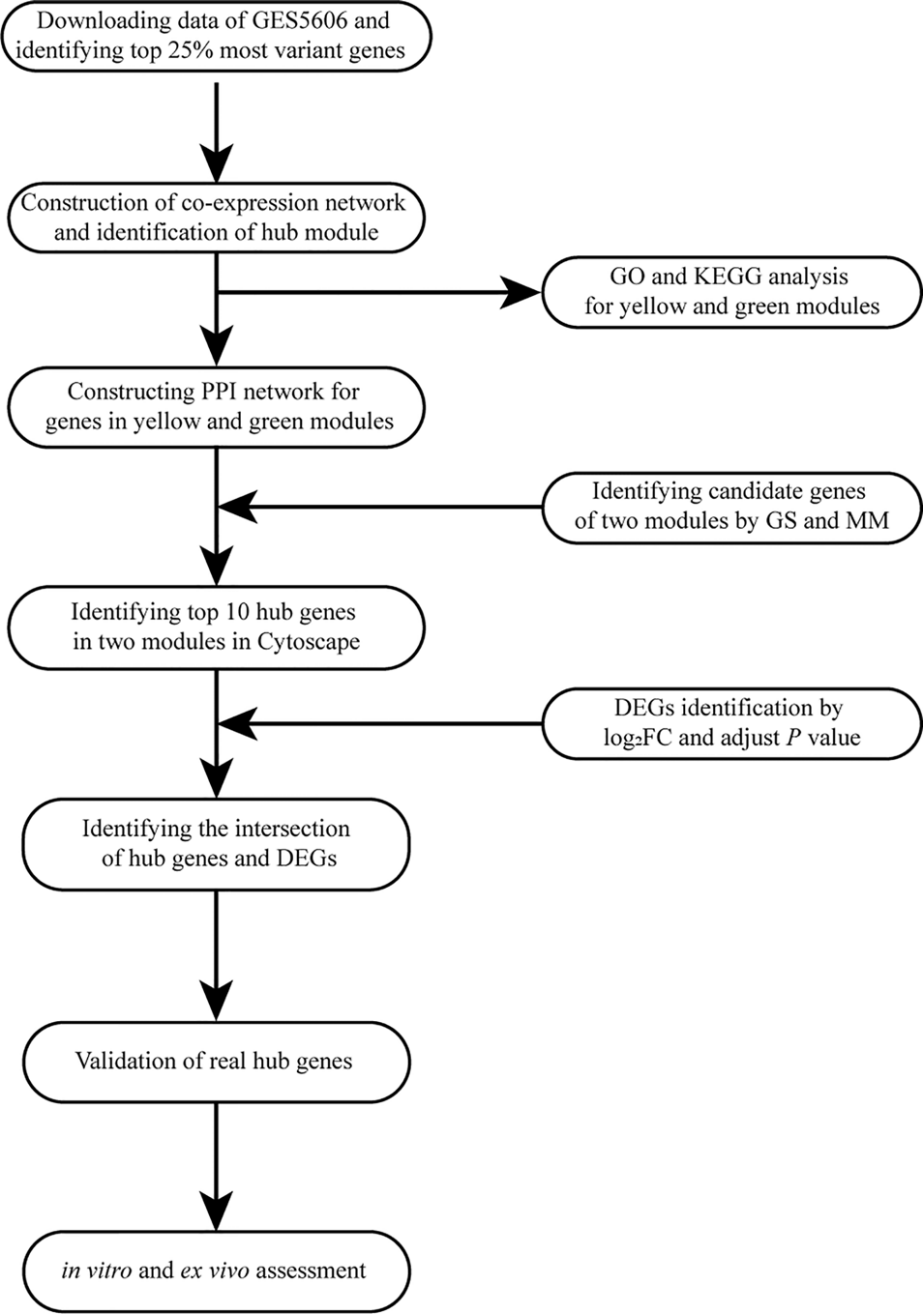
Flow diagram of the analysis procedure: data collection, preprocessing, analysis, and validation. GO, Gene Ontology; KEGG, Kyoto Encyclopedia of Genes and Genomes; PPI, protein-protein interaction; GS, gene significance; MM, module membership; DEG, differentially expressed gene; FC, fold change.

A workflow of this study is shown in **Figure 1**. We downloaded the raw microarray data of GSE5606 from the Gene Expression Omnibus (GEO) database (http://www.ncbi.nlm.nih.gov/geo/). Dataset GSE5606 was performed on GPL1355 [Rat230_2] Affymetrix Rat Genome 230 2.0 Array. The overall design was as follows: male wistars were anesthetized (Halothane 2–5%) and randomly received a tail vein injection of either saline or STZ 60 mg/kg. Animals were recovered and housed in pairs on fibercycle bedding (12 h light:dark cycle, 50–70% humidity, 19–21°C) for the duration of the study. Animal weight and blood glucose were recorded weekly. After 16 weeks the whole heart was excised from the animal after cervical dislocation and perfused with 60 ml of DEPC treated PBS pH 7.4 at 15 ml/min. LV was dissected away and stored in RNAlater (Qiagen) at −80°C. RNA from seven animals from each of the two groups was hybridized to an Affymetrix Rat Genome 230 2.0 Genechip, and the resulting changes in gene expression were analyzed.

The Robust Multi-array Average (RMA) algorithm in the “Affy” package under the R environment was used to preprocess the raw data (excel files) (11). After background correction, quantile normalization, and probe summarization, the top 25% of most variant genes by analysis of variance (3,601 genes) were selected for WGCNA analysis.

### Co-Expression Network Construction

R package WGCNA was adopted to conduct the co-expression network for the genes mentioned above (10). The weighted adjacency matrix was constructed with the formula a_mn_=|c_mn_|^β^ (a_mn_=adjacency between gene m and gene n, c_mn_=Pearson’s correlation between gene m and gene n). Parameter β is a soft-threshold that could emphasize strong correlations between genes and penalize weak correlations. Then, the weighted adjacency matrix was transformed into a topological overlap measure (TOM) matrix, which could measure the network connectivity of a gene defined as the sum of its adjacency with all other genes for the network generation. According to the TOM-based dissimilarity (1-TOM) measure with a minimum size of 30 for the gene dendrogram, average linkage hierarchical clustering was conducted, and genes with similar expression profiles were classified into the same gene modules. The branches of the tree labeled with a specific color represented one module containing highly correlated genes. The gray section donated background genes that belonged to none of the modules.

### Identification of Significant Modules

Two ways were applied to identify modules related to DbCM. We first defined module eigengenes (MEs) as the major component in the principal component analysis to summarize the expression pattern of all genes in a given module into a single characteristic expression profile. The correlation between MEs and sample traits was calculated to identify the significant module. In addition, the gene significance (GS) was defined as the log10 transformation of the *P*-value (GS=lg*P*) in the linear regression between gene expression and sample information. Then, module significance (MS) was defined as the average GS for all the genes involved in the module. Finally, the module with the absolute MS ranked first among all the selected modules was considered as the one related to the sample traits.

### Functional Enrichment Analysis

The Database for Annotation, Visualization, and Integrated Discovery (DAVID) is a web-based analytical tool (https://david.ncifcrf.gov/) (12, 13), which can provide functional interpretation of genes derived from genomic studies. We uploaded all genes in the hub module into DAVID for Gene Ontology (GO) and Kyoto Encyclopedia of Genes and Genomes (KEGG) pathway enrichment analysis. The GO terms of biological processes (BP), molecular functions (MF), and cellular components (CC) were respectively assessed. *P* < 0.05 was set as the cut-off criteria. Finally, we used the R to visualize the results above.

### Identification of Hub Genes

After the most significant module was identified, hub genes were initially screened according to the criteria that GS > 0.9 and Module Membership (MM) > 0.8. Then, we uploaded the selected genes in the hub module to the Search Tool for the Retrieval of Interacting Genes (STRING) database (14, 15) and used the Cytoscape software to visualize the protein-protein interaction (PPI) network. The “cytoHubba” plugin in Cytoscape was used to select the top hub genes in the PPI network.

### Validation of Hub Genes

The “Limma” R package was conducted to recognize differentially expressed genes (DEGs) between the DbCM and normal samples. *P*-values were adjusted to control the false discovery rate caused by multiple testing. Then, adjusted *P*-value ≤ 0.05 and |log_2_ fold change (FC)|≥1.2 were set for up- and downregulated genes. Volcano plot and hierarchical clustering analysis were carried out by R packages ggplot2 and pheatmap, respectively. Venn diagram was performed by online tool jvenn (http://jvenn.toulouse.inra.fr/app/example.html) (16) to overlap the hub genes in the DEGs above. The overlapped hub genes were chosen to be further analyzed and validated by the following experiments.

### Experimental Diabetic Mouse Model

All animal studies were conducted in conformity to a protocol confirmed by the Institutional Animal Care and Use Committee of Tongji Medical College, Huazhong University of Science and Technology. Male C57BL/6J mice (8 weeks old) were purchased from Beijing Vital River Laboratory Animal Technology Co., Ltd (Beijing, China). Type 1 diabetes was induced in C57BL/6J mice by intraperitoneal injections of streptozotocin (STZ, Cat#S0130, Sigma-Aldrich) 60 mg/kg dissolved in 0.1 mol/L citrate buffer (pH 4.5) for 5 consecutive days. Tail vein blood glucose was measured *via* glucose sticks (One-touch Ultra) on the seventh day after the last STZ injection. Mice were considered diabetic and enrolled in the study when random blood glucose was >16.7 mmol/L. Body weight and blood glucose were monitored periodically, and insulin lantus would be administrated subcutaneously when blood glucose went higher than 27.7 mmol/L. The control mice were intraperitoneally injected with the same dosage of citrate buffer. All mice were fed for 26 weeks and sacrificed at the age of 34 weeks with excessive xylazine and ketamine mixture (17).

### Echocardiography Analysis

Mice were anesthetized with inhalational isoflurane (1–2%), and echocardiography using a high-resolution imaging system with a 13-MHz linear ultrasound transducer (VisualSonics Vevo770, VisualSonics Inc., Toronto,Canada). And data were then analyzed with its supporting software packages (18).

### Reverse-Transcriptional Quantitative Polymerase Chain Reaction

Total RNA was extracted from cardiac tissues with TRIzol reagent (Takara, Kyoto, Japan). The RNA was reverse-transcribed into complementary DNA using the Prime Script RT reagent Kit with gDNA Eraser (Takara, Kyoto, Japan). qPCR was conducted with 2X Universal SYBR Green Fast qPCR Mix (Abclonal, Wuhan, China) and Step-One Real-time PCR systems (Applied Biosystems, CA, USA). The relative gene expression was calculated using the 2^−ΔΔCt^ method and normalized to the reference Glyceraldehyde 3-phosphate dehydrogenase (GAPDH). Primers of selected genes are available in **Table 1**.

**Table 1:** Primers of genes determined by quantitative real-time polymerase chain reaction.

Gene symbol	Species	Forward primer	Reverse primer
ANGPTL4	Mouse	GGGACTGCCAGGAACTCTTC	GAAGTCCACAGAGCCGTTCA
ACOT1	Mouse	AGTGCTGATTCAAGGGCTGG	TTCTCGCAGCTGGATTGAAC
HMGCS2	Mouse	GAAGAGAGCGATGCAGGAAAC	GTCCACATATTGGGCTGGAAA
PDK4	Mouse	AGGGAGGTCGAGCTGTTCTC	GGAGTGTTCACTAAGCGGTCA
DECR1	Mouse	GATCCGGGTCCTCAGAGGTTT	ATCAGGTGGTAGCATAGGCTT
SLC2A4	Mouse	GTGACTGGAACACTGGTCCTA	CCAGCCACGTTGCATTGTAG
HK2	Mouse	TGATCGCCTGCTTATTCACGG	AACCGCCTAGAAATCTCCAGA

### Western Blotting

Tissues or cultured cells were lysed in RIPA lysis buffer containing protease and phosphatase inhibitor cocktail (MedChemExpress, NJ, USA). Protein concentration was determined by BCA assay (Boster, CA, USA). Equal amounts of protein were resolved by 10% SDS-PAGE gels and transferred to PVDF membranes (Merck Millipore, Tullagreen, Carrigtwohill, County Cork, Ireland). Membranes were blocked for 1 h in 5% bovine albumin (BSA), incubated with primary antibodies against FAK (1:1,000, Cat# 3285S, Cell Signaling Technology), p-FAK (1:1,000, Cat# 8556S, Cell Signaling Technology), cleaved caspase 3 (1:1,000, Cat# 9661S, Cell Signaling Technology), Bax (1:1,000, Cat# 2772S, Cell Signaling Technology), Ac-SOD2 (1:1,000, Cat# AF4360, Affinity), SOD2 (1:1,000, Cat# bs-20668R, Bioss), SIRT3 (1:1,000, Cat# A5718, Abclonal), Bcl-2 (1:1,000, Cat# A0208, Abclonal), NOXA2/P67phox (1:1,000, Cat# A1178, Abclonal), NOX2/gp91phox (1:1,000, Cat# A1636, Abclonal), NCF1/P47phox (1:1,000, Cat# A1148, Abclonal), ANGPTL4 (1:1,000, Cat#CSB-PA005044, Cusabio), GAPDH (1:5,000, Cat# CSB-MA000071M1m, Cusabio), β-actin (1:5,000, Cat#66009-1-Ig, Proteintech), or β-tubulin (1:1,000, Cat#GB11017B, Servicebio) at 4°C overnight. Afterward, the membranes were incubated with HRP-conjugated anti-rabbit IgG (Cat#111-035-003, Jackson ImmunoResearch, West Grove, USA) or anti-mouse IgG (Cat#115-035-003, Jackson ImmunoResearch, West Grove, USA) secondary antibodies for 1 h at room temperature. The results were visualized on Tanon-5200 Chemiluminescent Imaging System (Tanon Science & Technology Co., Ltd).

### Histology and Immunohistochemistry

The mice cardiac tissues were fixed overnight in 4% paraformaldehyde and embedded in paraffin. Then we cut the tissues into 5-μm-thick slices, which were deparaffinized and subjected to antigen retrieval in hot citric acid buffer (PH 6.0) for 20 min. After cooling, slides were blocked with 5% BSA in TBST for 1 h and incubated overnight with primary antibodies against ANGPTL4 (1:100, Cat# A2011, Abclonal), NCF1/P47phox (1:100, Cat# Sc-14015, Santa Cruz Biotechnolog), and cleaved caspase 3 (1:100, Cat# 9661S, Cell Signaling Technology) at 4°C overnight, followed by secondary antibodies (1:200) for 30 min at room temperature. Staining was visualized by 3,3-diaminobenzidine (Vector Laboratories, Burlington, CA, USA) and hematoxylin under the microscope (Olympus BX61, Tokyo, Japan). Hematoxylin and eosin (H&E) and wheat germ agglutinin (WGA) (Cat# L4895, Sigma-Aldrich) staining for cardiomyocyte area assessment were performed as previously described (18). The outline of myocytes was traced using Image J software to determine myocyte cross-sectional area in the left ventricle of each animal. A value from each heart was calculated by measuring about 400–600 cells in a remote area from five randomly selected image areas in an individual heart.

### Cell Culture and Treatment

Human cardiomyocytes (AC16) were purchased from Sciencell (Cat# 6200, San Diego, CA, USA). Cells were carefully cultured according to product instruction. Cells were cultured in DMEM (normal glucose, 5.5mM-glucose) supplemented with 10% fetal bovine serum (Gibco, CA, USA) and 1% penicillin-streptomycin antibiotics (Life Technologies). High glucose (HG) culture media was made by supplementing additional D-glucose (Cat#G7021, Sigma-Aldrich) for a final D-glucose concentration at 30 mM.

Specific small interfering RNA (siRNA) was designed and provided by Guangzhou RiboBio Co., Ltd (Guangzhou, China). The AC16 cells were seeded in six-well plates and then transfected with 100 nM siRNA targeting ANGPTL4 (si-ANGPTL4) or negative control siRNA (si-NC) using HighGene infection reagent (Cat# RM09014, Abclonal, Wuhan, China) according to the manufacturer’s instructions. Another batch of cells were treated with recombinant human ANGPTL4 protein purchased from Abclonal (Cat# RP02179).

### Immunofluorescence

Heart sections were deparaffinized and subjected to antigen retrieval in hot citric acid buffer (PH 6.0) for 20 min. After cooling, slides were blocked with 5% BSA in TBST for 1 h and incubated overnight with primary antibodies against ANGPTL4 (1:100, Cat# A13425, Abclonal) at 4°C overnight, followed by incubation with a goat anti-rabbit-Cy3 (1:200, Cat# GB21303, Servicebio) for 1 h at room temperature. Sections were washed with PBS three times and then counterstained with 4’,6-diamidino-2-phenylindole fluorescent dye (DAPI). While the AC16 cells were fixed with 4% paraformaldehyde for 10 min, then washed with PBS three times. The cells were blocked with normal goat serum and incubated with anti- ANGPTL4 (1:100, Cat# A13425, Abclonal) antibody at 4°C overnight. Cells were washed with PBS three times and then incubated with a goat anti-rabbit-Cy3 (1:200, Cat# GB21303, Servicebio) or anti-rabbit-FITC (1:200, Cat# GB22303, Servicebio) for 1 h. Cells were washed with PBS three times and then counterstained with DAPI. Digital images were observed under a microscope with SOPTOP ICX41 or OLYMPUS.

### TUNEL and Dihydroethidium Staining

According to the instructions for the One Step TUNEL Apoptosis Assay Kit (Cat# C1088 Beyotime, Shanghai, China), heart sections and AC16 cells were treated with terminal deoxynucleotidyl TUNEL staining. All the nuclei were counterstained with DAPI. We calculated the ratio of TUNEL-positive nuclei and the total number of nuclei to define the percentage of apoptotic cells. The production of intracellular ROS was analyzed with Dihydroethidium (DHE, Cat# S0063, Beyotime, Shanghai, China), respectively, according to the manufacturer’s instructions. Briefly, treated cells or fresh frozen sections were washed with PBS, and then ROS-capturing reagents were added and incubated at 37°C in the dark for 30 min and visualized by fluorescence microscopy.

### Statistical Analysis of Experimental Data

Data were presented as the mean ± standard deviation (SD). Statistical analysis was executed with GraphPad Prism (version 9.0, San Diego, CA, USA). Statistical analyses were performed with Student’s *t*-test. Differences at the 95% confidence level (*P* < 0.05) were considered statistically significant.

## Results

### Training Set Quality Assessment

**Figure 2:**
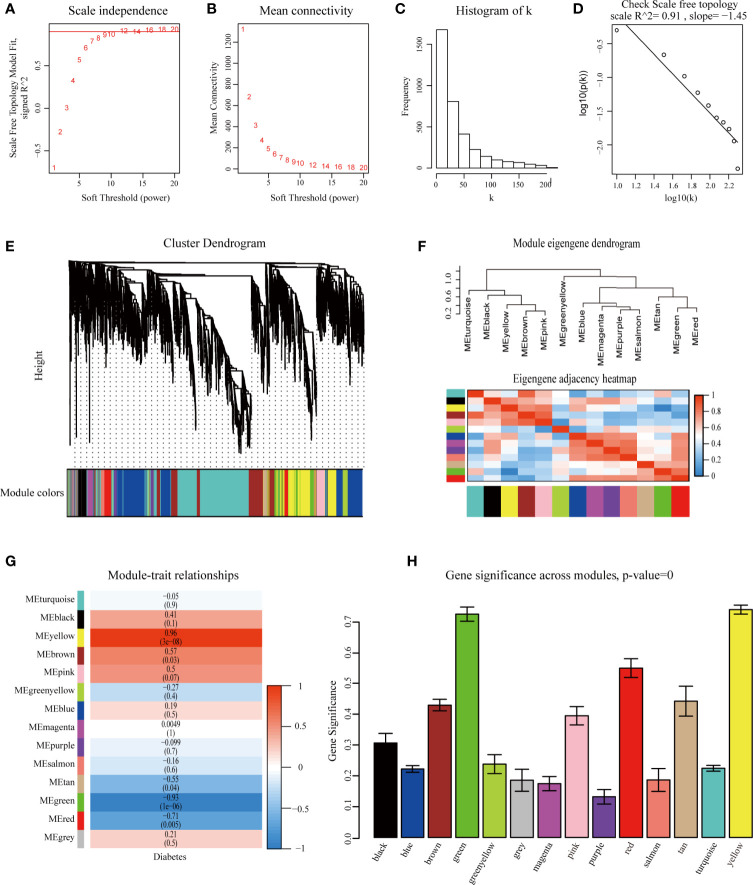
Identification of modules associated with diabetic cardiomyopathy using the weighted gene co-expression network analysis (WGCNA). **(A)** Analysis of the scale-free fit index for various soft-thresholding powers (β). **(B)** Analysis of the mean connectivity for various soft-thresholding powers. **(C)** Histogram of connectivity distribution when β=12. **(D)** Checking the scale-free topology when β=12. Identification of modules associated with diabetic cardiomyopathy. **(E)** Dendrogram of the top 25% most variant genes clustered based on a dissimilarity measure (1-TOM). **(F)** Module eigengene dendrogram and eigengene network heatmap summarize the modules yielded in the clustering analysis. **(G)** Heatmap of the correlation between module eigengenes and diabetic cardiomyopathy. **(H)** Distribution of average gene significance and errors in the modules associated with diabetic cardiomyopathy. TOM, Topological Overlap Matrix.

The overall flow diagram of our study is presented in **Figure 1**. In this study, no sample was removed in GSE5606 by the outliers check (**Supplementary Figure S1A**). Then, the top 25% of most variant genes by analysis of variance (3,601 genes) were chosen for subsequent WGCNA analysis. And, the soft-thresholding power of β =12 (scale-free R^2 =^ 0.91) was selected to ensure a scale-free network (**Figures 2A–D**).

### Weighted Co-Expression Network Construction and Key Modules Identification

WGCNA was performed to classify the genes with similar expression profiles into the same modules. A total of 14 modules were identified *via* the average linkage clustering (**Figure 2E**). There were 111 genes in the black module, 767 genes in the blue module, 447 genes in the brown module, 182 genes in the green module, 66 genes in the green-yellow module, 39 genes in the gray module, 78 genes in the magenta module, 104 genes in the pink module, 74 genes in the purple module, 138 genes in the red module, 47 genes in the salmon module, 48 genes in the tan module, 1,067 genes in the turquoise module, and 433 genes in the yellow module. TOM plots were constructed to show the pairwise gene correlation within each module (**Supplementary Figure S1B**). Then we calculated eigengenes of all modules and clustered them conforming to their correlation. Module eigengene dendrogram showed that the 14 modules were mainly divided into two clusters, and similar results were demonstrated by the heatmap plotted (**Figure 2F**). Module eigengene was also analyzed for correlation with the disease trait. From the heatmap of module-trait correlation, we identified that the yellow and green modules were the most highly correlated with the disease trait (**Figure 2G**). **Figure 2H** illustrated the gene significance of each module with DbCM phenotype, and the most significant ones were also yellow and green modules. We recognized the yellow and green modules as the most significant modules for further analysis based on the two methods above.

### Hub Gene Identification in Selected Modules

**Figure 3:**
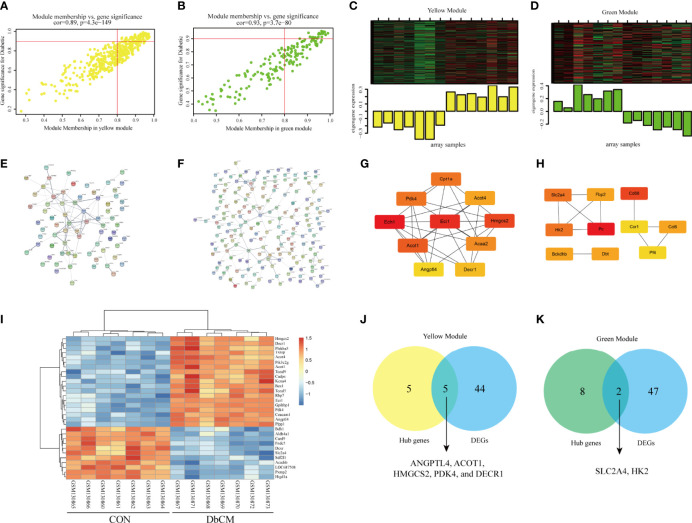
Validation of real hub genes in yellow and green modules. **(A, B)** Scatter plot of module eigengenes in the yellow module and the green module. **(C, D)** The module heatmap and eigengene of array samples in the yellow module and the green module. **(E, F)** PPI network analysis of representative genes in the yellow module and the green module. **(G)** Top 10 hub genes in the yellow module selected by the “cytoHubba” plugin in the Cytoscape based on the MCC algorithm. **(H)** Top 10 hub genes in green model selected by the “cytoHubba” plugin in the Cytoscape based on MCC algorithm. **(I)** Heatmap of top 30 DEGs between normal and diabetic animals heart tissue. **(J, K)** Identification of the intersection between DEGs and the hub genes in the yellow module and the green module. DEG, differentially expressed gene; PPI, protein-protein interaction.

**Figures 3A, B** illustrated the correlation between MM and GS in the yellow and green modules. **Figures 3C, D** displayed the module heatmap and the eigengene. In the diabetic heart samples, the eigengene expression of the yellow module was higher than expression in the normal heart samples. The opposite result was observed in the green module. Initially, candidate hub genes were initially screened according to the criteria (GS > 0.9 and MM > 0.8). A total of 74 genes that were highly connective to the yellow module were identified as candidate hub genes. Then, we constructed a PPI network for candidate hub genes in the yellow module according to the STRING database, and a representative local network was shown in **Figure 3E**. The top 10 hub genes were selected by the “cytoHubba” plugin in the Cytoscape based on the MCC algorithm (**Figure 3G**). Under the threshold adjusted *P*-value ≤ 0.05 and |log_2_ FC|≥1.2, a total of 49 DEGs (29 upregulated and 20 downregulated) were chosen for subsequent analysis. **Figure 3I** present the representative heatmap of the top 30 DEGs. The volcano plot of all DEGs was shown in **Supplementary Figure S1E**. Eventually, the common genes between the top 10 hub genes and DEGs were screened as the final candidates to be further analyzed and validated, including *ANGPTL4*, *ACOT1*, *HMGCS2*, *PDK4*, and *DECR1* (**Figure 3J**). With regard to the green module, highly connective genes (28 genes) were identified as candidate hub genes. For the limited selected genes in this module, we uploaded all genes in the module to construct the PPI network, and a representative local network was shown in **Figure 3F**. We selected the top 10 hub genes by the same method above (**Figure 3H**). Finally, the common genes between top 10 genes and DEGs were screened as the final candidates. Only two genes, *SLC2A4* and *HK2*, satisfied the above conditions (**Figure 3K**). More details about the seven hub genes were displayed in **Table 2**. All genes of the PPI network among yellow and green modules were provided in **Supplementary Figures S3C, D**.

**Table 2:** The selected hub genes in yellow and green modules.

Module	Gene symbol	Full name	log_2_ fold change	Adjust *P* value	Regulation
Yellow	ANGPTL4	Angiopoietin-like 4	4.595	3.88E-09	Up
Yellow	ACOT1	Acyl-CoA thioesterase1	4.120	3.88E-09	Up
Yellow	HMGCS2	3-hydroxy-3-methylglutaryl-CoenzymeA Synthase 2	3.562	8.07E-08	Up
Yellow	PDK4	Pyruvate Dehydrogenase kinase 4	2.178	8.07E-08	Up
Yellow	DECR1	mitochondrial 2,4-dienoyl-CoA reductase 1	1.251	1.29E-07	Up
Green	SLC2A4	Solute carrier family 2 (facilitated glucose transporter), member 4	−1.956	2.35E-06	Down
Green	HK2	Hexokinase 2	−1.776	2.24E-05	Down

### Functional Enrichment Analysis

**Figure 4:**
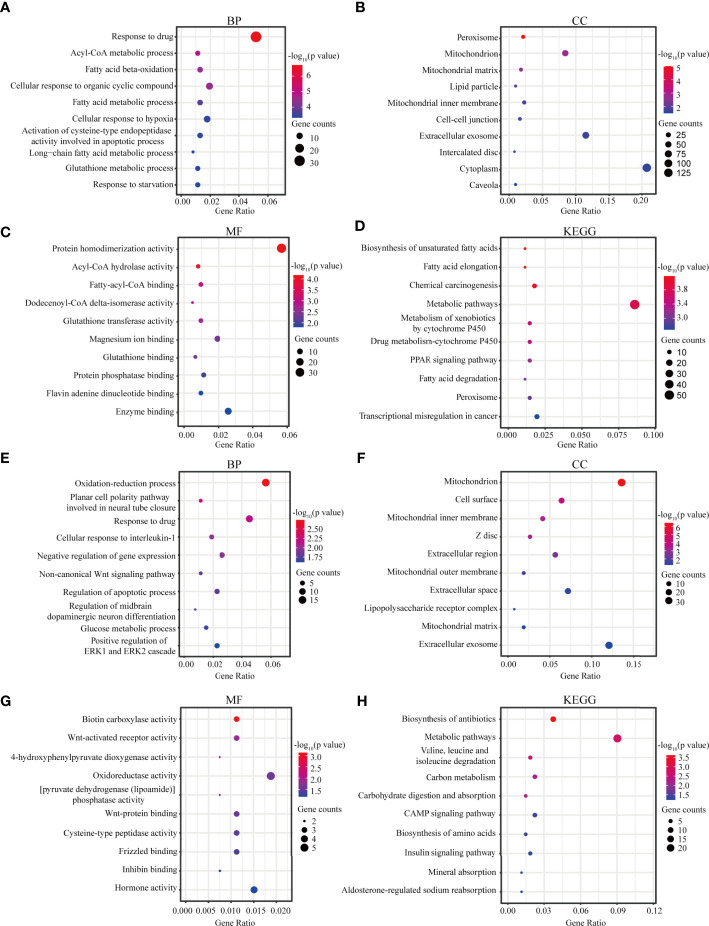
Functional analysis. **(A–C)** Biological process, Cellular component, and Molecular function GO terms for genes in the yellow module. **(D)** KEGG analysis for genes in the yellow module. **(E–G)** Biological process, Cellular component, and Molecular function GO terms for genes in the green module. **(H)** KEGG analysis for genes in the green module. GO, Gene Ontology; KEGG, Kyoto Encyclopedia of Genes and Genomes; BP, biological process; CC, cellular component; MF, molecular function.

The most significant GO term and KEGG pathways of all genes in the yellow module are shown in **Figures 4A–D**. Among the biological processes, the cluster was significantly associated with response to drug, Acyl-CoA metabolic process, and fatty acid beta-oxidation (**Figure 4A**). In cellular component enrichment analysis, the terms of the peroxisome and mitochondrion were enriched (**Figure 4B**). Among the molecular function analysis, our results showed that the genes were significantly associated with the protein homodimerization activity, Acyl-CoA hydrolase activity, and fatty-acyl-CoA binding (**Figure 4C**). As to KEGG pathway analysis, metabolic pathways, biosynthesis of unsaturated fatty acids, and chemical carcinogenesis were mainly associated with these genes (**Figure 4D**). **Figures 4E–H** displayed the most significant GO term and KEGG pathways of all genes in the green module. GO analysis results showed that the oxidation-reduction process, mitochondrion, and biotin carboxylase activity were significantly enriched (**Figures 4E–G**). Metabolic pathways and biosynthesis of antibiotics were most enriched in KEGG pathways analysis (**Figure 4H**).

### Diabetic Mice Displayed Cardiac Function Impairment and Left Ventricular Hypertrophy

**Figure 5:**
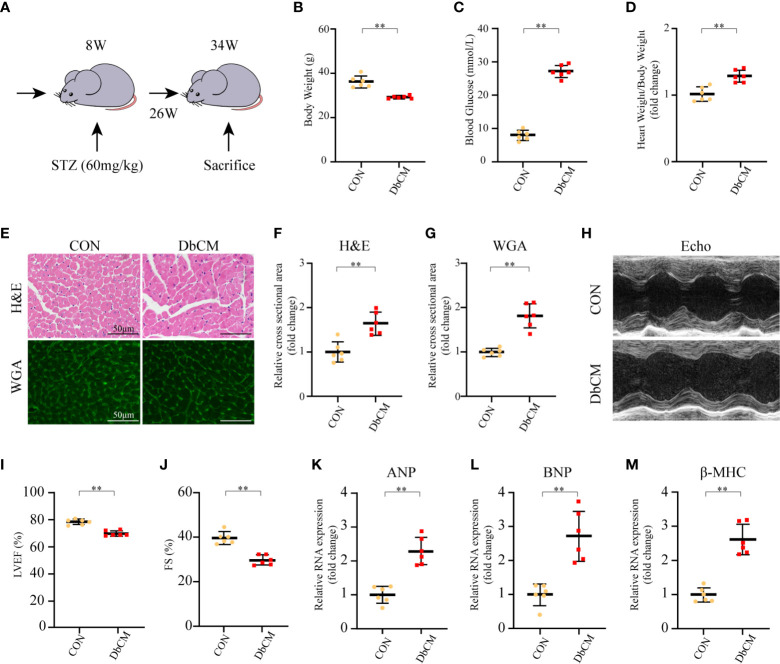
Establishment of diabetic cardiomyopathy mouse model. **(A)** Schematic illustration of diabetic cardiomyopathy mouse model. **(B–D)** Body weight, random blood glucose, and heart weight/body weight at 26 weeks after STZ injection (n=6 each). **(E)** Representative images of H&E staining and WGA staining in the hearts of CON and DbCM mice (n=6; scale bar, 50 μm). **(F)** Statistical results of the myocyte cross-sectional areas of H&E staining (n=6, at least 100 cells per mouse were analyzed). **(G)** Quantitative analysis of myocyte cross-sectional areas of WGA staining (n=6, at least 100 cells per mouse were analyzed). **(H)** Representative echocardiographic M-mode images. **(I)** Echocardiographic measurements of LVEF. **(J)** Echocardiographic measurements of FS. **(K–M)** Representative RNA expression of *ANP*, *BNP*, and *β-MHC*, respectively. Data are shown as mean ± SD (n=6 each). *p < 0.05, **p < 0.01 *vs* CON group (student *t*-test). CON, control mice without diabetes; DbCM, diabetic cardiomyopathy; STZ, streptozotocin; H&E, hematoxylin eosin; WGA, wheat germ agglutinin; LVEF, left ventricle ejection fraction; FS, fractional shortening; ANP, atrial natriuretic peptide; BNP, B type natriuretic peptide; β-MHC, β-myosin heavy chain.

As described in *Materials and Methods*, we used a mouse model of persistent hyperglycemia induced by STZ, reflecting insulinopenic type 1 DM (**Figure 5A**). After STZ injection, random blood glucose and body weight were measured. The diabetic mice exhibited a significant reduction in body weight compared with the control group at 26 weeks after STZ injection (**Figure 5B**). Random blood glucose and heart weight/body weight were significantly increased in diabetic group compared with the control group (**Figures 5C, D**). Diabetic mice also displayed a significant increase in 24h urine volume (**Supplementary Figure S2**). The myocardial structure was examined by H&E and WGA staining (**Figure 5E**). Diabetic mice had a significant larger cardiomyocyte transverse cross-sectional area (**Figures 5E–G**). To evaluate the changes of left ventricular performance in diabetic mice, M-mode echocardiography was performed at 26 weeks after STZ injection (**Figure 5H**). Compared to control mice, diabetic mice showed a significant reduction of ejection fraction and fraction shorting (**Figures 5H–J**). As expected, these pathological provocations led to a significant elevations in atrial natriuretic peptide (ANP), B type natriuretic peptide (BNP), and β-myosin heavy chain (β-MHC) mRNA expression (**Figures 5K–M**). These data indicated that the type 1 DbCM was successfully established, characterized by diastolic and systolic dysfunction and myocardial hypertrophy.

### Experimental Validations of Hub Genes

**Figure 6:**
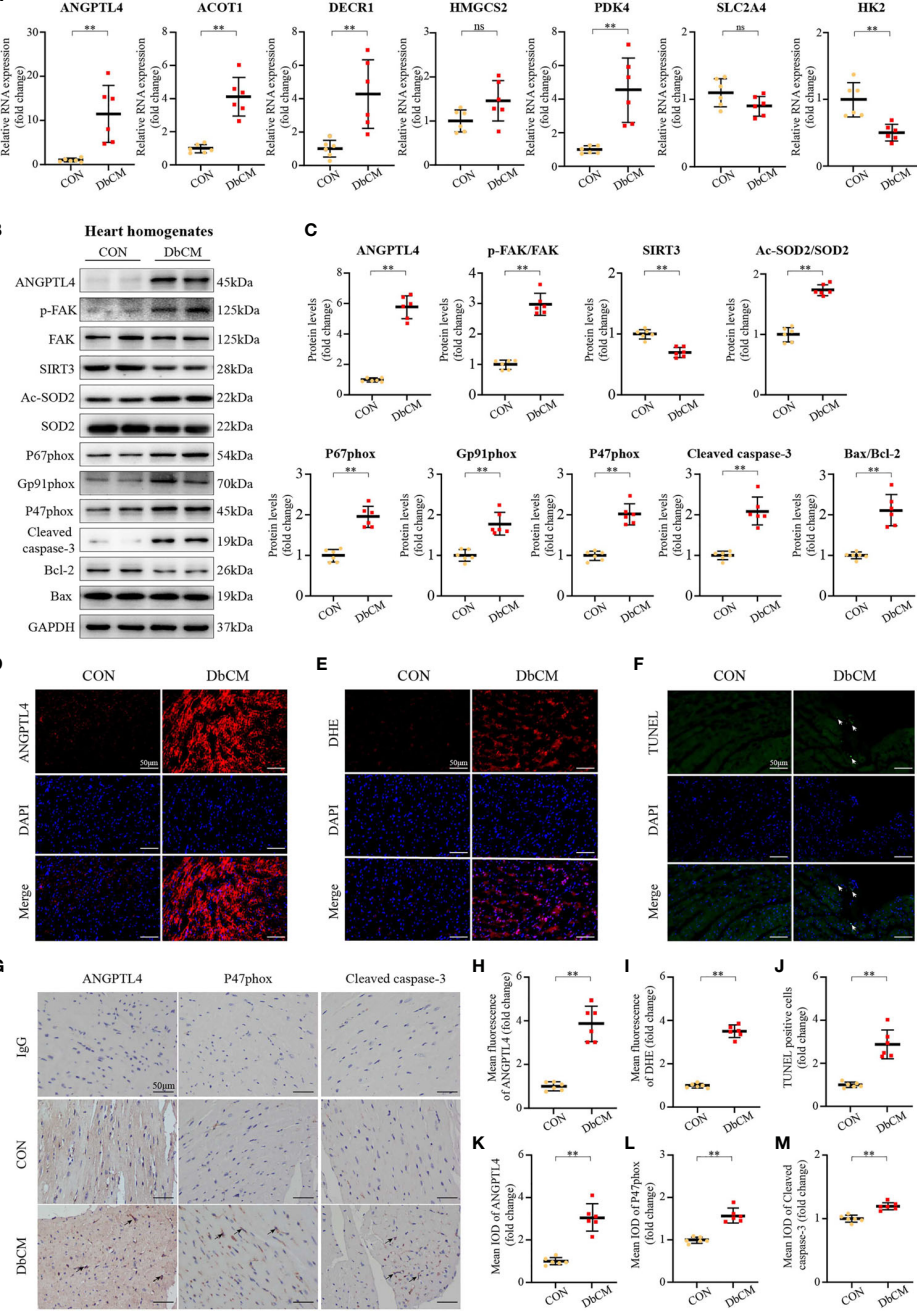
Screening of a novel therapeutic target for DbCM. **(A)** Relative mRNA expression of hub genes normalized to a GAPDH internal control (n=6 each). Values are expressed relative to control groups. **(B, C)** Representative Western blots and analysis of ANGPTL4 expression; p-FAK(Y397)/FAK ratio; SIRT3 expression; Ac-SOD2/SOD2 ratio; P67phox expression; Gp91phox expression; P47phox expression; Cleaved caspase-3 expression; Bcl-2 expression; Bax expression. Equal protein loading was confirmed using an anti-GAPDH antibody (n=6 each). **(D)** Representative images of ANGPTL4 fluorescence in heart sections. Scale bar, 50 μm. **(E)** Representative images of DHE fluorescence in heart sections. Scale bar, 50 μm. **(F)** Representative images of apoptotic cardiomyocytes (200×). The apoptotic cells were detected by TUNEL (green), and the nuclei were detected by DAPI (blue). Scale bar, 50 μm. **(G)** Representative immunohistochemical stainings of ANGPTL4, P47phox, and Cleaved caspase-3 (n=6 each). Scale bars, 50 μm. **(H)** Quantification of ANGPTL4 fluorescence intensity in heart sections (n=6 each). **(I)** Quantification of DHE fluorescence intensity in heart sections (n=6). **(J)** Percentage of TUNEL positive cells. **(K–M)** Representative immunohistochemical quantification of ANGPTL4, P47phox, and Cleaved caspase-3. Scale bars, 50 μm. Data are shown as mean ± SD (n=6 each); *p < 0.05, **p < 0.01 *vs* CON group (student *t*-test). ns represents statically non-significant; IOD, the integral optical density.

Reverse-transcriptional quantitative polymerase chain reaction showed that the mRNA expression levels of *ANGPTL4*, *ACOT1*, *DECR1*, *PDK4*, and *HK2* were significantly different in diabetic hearts contrast to the normal controls excluding *HMGCS2* and *SLC2A4* (**Figure 6A**). Indeed, we found an 11.5-fold increase in *ANGPTL4* level in the DbCM group compared with the control group, which might play an essential role in the development of DbCM. Consistently, western blot (**Figures 6B, C**), immunofluorescence (**Figures 6D, H**), and immunohistochemistry (**Figures 6G, K**) results also indicated that the amount of ANGPTL4 was significantly upregulated in the diabetic hearts compared with the normal controls.

### ANGPTL4-Mediated Oxidase Pathway in DbCM

ANGPTL4 is an endogenous protein with a C-terminal fibrinogen-like globular domain (cANGPTL4) and an N-terminal coiled-coil domain connected by the linker region (nANGPTL4), which is associated with vascular permeability, inflammation, oxidative stress, angiogenesis, wound healing, and lipid metabolism (19, 20). Cardiac-restricted overexpression of ANGPTL4 in mouse model significantly inhibits cardiac LPL activity and results in cardiomyopathy (21). Recent studies have indicated that ANGPTL4 is also involved in the regulation of oxidative stress signaling. Zhu et al. (22) demonstrated that tumor-derived ANGPTL4 interacted with integrins to stimulate NADPH oxidase-dependent production of superoxide $\left({\text{O}}_{2}^{-\cdot }\right)$ in tumor cells. A TAZ-ANGPTL4-NOX2 axis regulates ferroptotic cell death and chemoresistance in epithelial ovarian cancer (23). Thus, we hypothesize that ANGPTL4 could regulate DbCM *via* integrin/oxidative stress/apoptosis signaling pathway. Indeed, western blot analysis of heart homogenates showed that DbCM group, compared with the control group, had increased phosphorylation of focal adhesion kinase (p-FAK/FAK), an important effector downstream of integrins (24), upregulated NADPH oxidase activation (NOXA2/P67phox, NOXA/gp91phox, and NCF-1/P47phox), and enhanced apoptosis in the diabetic heart, as indicated by an increased level of cleaved caspase-3 and Bax/Bcl-2 (**Figures 6B, C**). Consistent with the above findings, increased DHE staining and P47phox immunohistochemistry results were also observed (**Figures 6E, G, I, L**). Furthermore, TUNEL staining and cleaved caspsase-3 immunohistochemistry proved increased apoptosis (**Figures 6F, G, J, M**). However, how integrin signaling was bridged to oxidative stress and apoptosis remains a puzzle.

Sirtuin-3(SIRT3), which belongs to the sirtuin family of nicotinamide adenine dinucleotide (NAD^+^)-dependent deacetylase, is expressed in a variety of tissues and is highly expressed in the heart (25, 26). SIRT3 regulates various cellular biological activities such as mitochondrial dynamics, metabolism, and cellular responses to oxidative stress (27). Intriguingly, SIRT3-mediated deacetylation is critical for the activity of superoxide dismutase 2 (SOD2), a mitochondrial enzyme that prevents vascular oxidative stress (28). Moreover, SIRT3/SOD2 signaling pathway has been shown to be involved in integrin-induced ROS generation in platelets (29) and tumor cells (30, 31). Therefore, we speculate that SIRT3 could be the missing intermediate between integrin and oxidative stress, and apoptosis. Indeed, opposite to the upregulated p-FAK/FAK ratio, SIRT3 expression was significantly decreased in the DbCM group, which resulted in deacetylation of SOD2, a key molecule involved in regulating ROS homeostasis (**Figures 6B, C**). Thus, these results suggest that ANGPTL4 could promote cardiac apoptosis *via* a FAK/SIRT3/ROS signaling pathway in DbCM.

### ANGPTL4 Induces Apoptosis *via* FAK/SIRT3/ROS Pathway in Human Cardiomyocytes Under High Glucose Condition *In Vitro*

**Figure 7:**
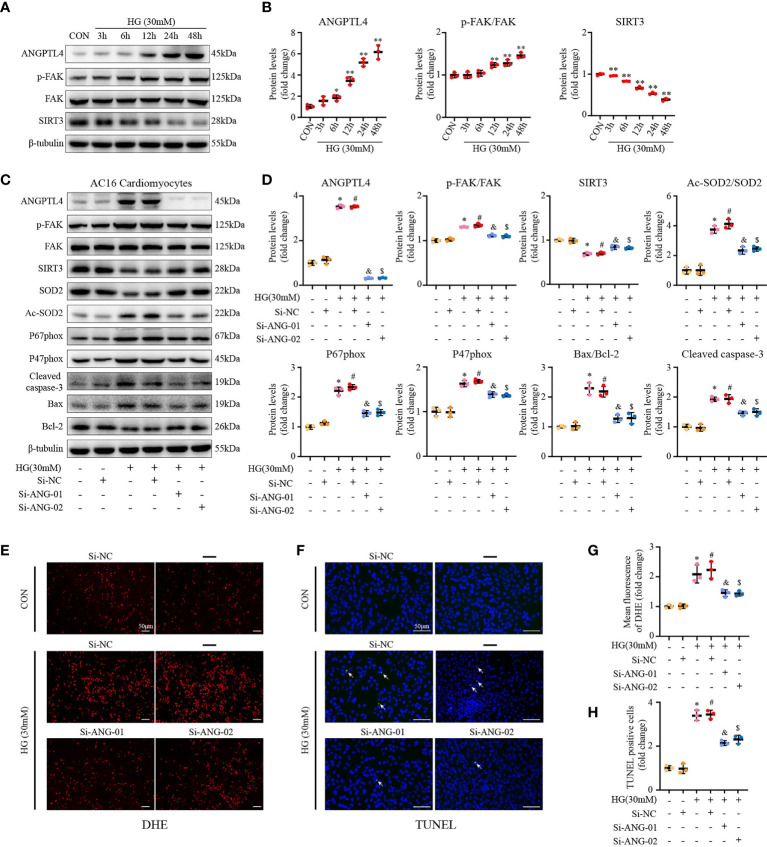
ANGPTL4 siRNA exhibited the anti-apoptotic and anti-oxidative effects in HG-treated AC16 cardiomyocytes. **(A)** Representative Western blots showing ANGPTL4, p-FAK(Y397), FAK, and SIRT3 levels in AC16 cardiomyocytes at indicated time points after treatment with HG (30 mM). **(B)** WB analysis of ANGPTL4 expression, p-FAK(Y397)/FAK ratio, and SIRT3 expression at indicated time points, respectively. **(C, D)** Representative Western blots and quantification of p-FAK(Y397), FAK, SIRT3, Ac-SOD2, SOD2, P67phox, P47phox, Cleaved caspase-3, Bcl-2, and Bax in cultured AC16 cardiomyocytes under the control or HG (30 mM) condition plus treatment with si-RNA targeting ANGPTL4 (si-ANGPTL4) or negative control siRNA (si-NC). Equal protein loading was confirmed using an anti-β-tubulin antibody. **(E, G)** Representative images and quantification of DHE fluorescence in cultured AC16 cardiomyocytes under the control or HG (30 mM) condition plus treatment with si-RNA targeting ANGPTL4 (si-ANGPTL4) or negative control siRNA (si-NC). Scale bars, 50 μm. **(F, H)** Representative images of apoptotic cardiomyocytes (200×) and percentage of TUNEL positive cells. The apoptotic cells were detected by TUNEL (green), and the nuclei were detected by DAPI (blue). Scale bar, 50 μm. Experiments were repeated at least three times independently with similar results. *p < 0.05, **p < 0.01 *vs* CON group. ^#^ *p* < 0.05 *vs* the CON+SiNC group; ^&^ *p* < 0.05 *vs* the HG+SiNC group; ^$^ *p* < 0.05 *vs* the HG+SiNC group (student *t*-test).

In order to further prove our hyothesis, *in vitro* cell culture studies were applied. Consistent with our *in vivo* findings, western blot results showed that high glucose (HG) could induce the expression of ANGPLT4 in culured human cardomyocytes (AC16) in a time-dependent manner, which peaked at 48 h (**Figures 7A, B**). Subsequent immunofluorescence staining of ANGPTL4 in AC16 further strengthened our above finding (**Supplementary Figure S3**). Moreover, we also detected increased Tyr397 phosphorylation of FAK and reduced expression of SIRT3 over time on HG treatment (**Figures 7A, B**). Simutanously, levels of p-FAK/FAK, NADPH oxidase (NOXA2/P67phox and NCF-1/P47phox), Ac-SOD2/SOD2, and apoptosis markers (Cleaved caspase-3 and Bax/Bcl-2) were significantly upregulated in AC16, while SIRT3 was downregulated (**Figures 7C, D**). As expected, these effects were abrogated by treatment of two independent siRNA for ANGPTL4, namely, si-ANG-01 and si-ANG-01 (**Figures 7C, D**). Besides, DHE and TUNEL staining results further reinforeced the above findings (**Figures 7E–H**).

**Figure 8:**
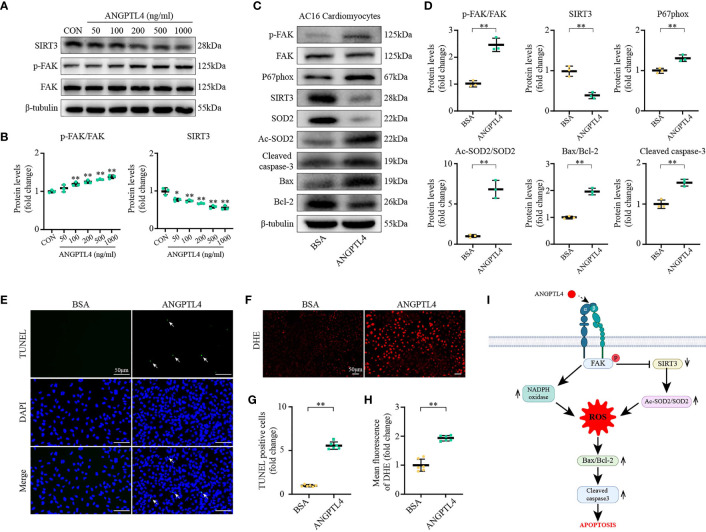
ANGPTL4 elevated SOD2 acetylation, oxidative stress, and apoptosis. **(A, B)** Representative immunoblots showing SIRT3, p-FAK(Y397), and FAK levels in AC16 cardiomyocytes at 48 h after different concentrations of ANGPTL4 treatment. **(C, D)** Representative Western blots and quantification of p-FAK(Y397), FAK, P67phox, SIRT3, SOD2,Ac-SOD2, Bax, Bcl-2, and Cleaved caspase-3 in AC16 cardiomyocytes after treatment with BSA or ANGPTL4 (200 ng/ml each) for 48 h. **(E, G)** Representative images of apoptotic cardiomyocytes (200×) after treatment with BSA or ANGPTL4 (200 ng/ml each) for 48 h and percentage of TUNEL positive cells. The apoptotic cells were detected by TUNEL (green), and the nuclei were detected by DAPI (blue). Scale bar, 50 μm. **(F, H)** Representative images and quantification of DHE fluorescence in AC16 cardiomyocytes after treatment with BSA or ANGPTL4 (200 ng/ml) for 48 h. Scale bar, 50 μm. **(I)** Schematic illustration shows ANGPTL4 interacting with integrin, inducing apoptosis in the Diabetic Cardiomyopathy *via* FAK/SIRT3/ROS pathway in cardiomyocyte. P represents phosphorylation, α and β represent subunit of the integrin. Data are shown as mean ± SD; *p < 0.05, **p < 0.01 vs BSA group (student t test).

On the other hand, the effect of exogenous recombinant human ANGPLT4 protein was tested in AC16 *in vitro*. We were able to show that exogenous ANGPLT4 treatment could dose dependently suppress the expression of SIRT3 and induce the expression of p-FAK(Tyr397) in AC16, which reach at a platform at around 200 ng/ml (**Figures 8A, B**). Meanwhile, other indices for FAK/SIRT3/ROS pathway **(**p-FAK/FAK, NOXA2/P67phox, NCF-1/P47phox, and Ac-SOD2/SOD2, DHE staining**)** and apoptosis (Cleaved caspase-3 and Bax/Bcl-2, TUNEL staining) were significantly increased (**Figures 8A–H**).

In conclusion, these results demonstrated that the yellow and green modules correlated the most with DbCM caculated by WGCNA, and ANGPTL4 was identified as one of the most significantly upregulated hub genes (*ANGPTL4*, *ACOT1*, *DECR1*, *HMGCS2*, and *PDK4*). Mechanistically, ANGPTL4 could promote cardiac apoptosis *via* a FAK/SIRT3/ROS-dependent signaling pathway in DbCM, as illustrated in **Figure 8I**.

## Discussion

In the present study, we constructed WGCNA to identify co-expression genes and possible biological pathways of DbCM based on the GSE5606 dataset. The yellow and green modules were most positively or negatively correlated with DbCM. The top hub genes, including *ANGPTL4*, *ACOT1*, *HMGCS2*, *PDK4*, *DECR1*, *SLC2A4*, and *HK2*, were identified and verified by RT-qPCR. The function analysis revealed that the metabolic and oxidation-reduction processes were significantly involved in DbCM formation and progression. We performed RT-qPCR to verify the top hub genes in the STZ-induced DbCM animal model, and ANGPTL4 expression was significantly elevated in the diabetic heart. Subsequently, we further investigated the role of ANGPTL4 in the process of DbCM *in vitro* and *ex vivo*. Here, we provide insight into the role of ANGPTL4 in non-metabolic effects. We show that ANGPTL4 may induce apoptosis in the DbCM heart *via* FAK/SIRT3/ROS pathway.

To the best of our knowledge, this is the first study to explore novel biomarkers and therapeutic targets for DbCM using WGCNA. After a series of bioinformatics analyses, seven hub genes, including *ANGPTL4*, *ACOT1*, *HMGCS2*, *PDK4*, *DECR1*, *SlC2A4*, and *HK2*, were especially outstanding. Some screened hub genes have been previously studied in DbCM. It was shown that ACOT1 played a crucial protective role in the diabetic heart *via* PPARα/PGC1α signaling (32). George et al. indicated that insulin-dependent diabetes induced the enzymes of ketone body synthesis in the heart, including HMGCS2, as well as increasing enzymes of fatty acid oxidation (33). The upregulation of PDK4 proved that the oxidation of glucose was minimized in diabetic animals through inhibition of pyruvate oxidation (33). During the development of diabetes, there is a shift in cardiac metabolism away from glucose metabolism towards fatty acid metabolism. This shift is generally associated with significant decreases in cardiac and muscle HK2 protein content (34). Diabetes also reduced the level of GLUT4 (encoded by *SLC2A4*) that functions as an insulin-regulated facilitative glucose transporter in the heart by 70% (35). However, after WGCNA analysis, the genes with similar expression profiles were sorted into the same modules, module eigengene was also analyzed for correlation with the disease trait, and the module with the highest correlation with the disease trait was selected for further analysis. Probably, the insulin pathway genes were not categorized in the selected modules; that is why they were not listed in the final hub genes. Whereas *SLC2A4* was indeed identified as a hub gene after bioinformatics analysis. However, in the validation stage, *SLC2A4* was downregulated in the STZ-induced diabetic heart when we compared with control group by RT-qPCR but remained statistically non-significant. This discrepancy may come from the limited number of the experimental animals or different experimental models. Thus, a larger sample and animal models other than STZ-induced type 1 diabetes, such as db/db or a combination of high-fat diet (42% energy intake from fat) and streptozotocin (three consecutive 55 mg/kg, body 52 weight, i.p.) induced type 2 diabetes (36), would be helpful to clarify the inconsistencies between *in silico* bioinformatic analysis and biological sample verification.

The human genetics studies showed that genetic inactivation of *ANGPTL4* improves glucose and lipid homeostasis and is associated with reduced risk of coronary artery disease and diabetes (37–39). Hyperglycemia is an important risk factor for DbCM in type 1 diabetic adolescent, leading to diastolic and systolic dysfunction (40, 41). The excess of glucose may deviate to deleterious metabolites and activation of pro-oxidative and inflammatory pathways, damaging pancreas and other peripheral tissue such as heart. In virtue of its ability to inhibit LPL, ANGPTL4 can play a vital role in lipid metabolism. Dyslipemia can enforce tissue damage and reduce its glucose assimilation. In consequence, increased oxidation, inflammation, and atherogenesis could lead to cardiovascular abnormalities and heart dysfunction (40). Oxidative stress has also been implicated in the onset and progression of cardiovascular diseases such as congestive heart failure and diabetes-associated heart dysfunction (42). Maintaining a LPL- ANGPTL4 balance is essential, and considering LPL and ANGPTL4 as cardiac specific biomarkers would be advantageous in detecting the stage of diabetes and in treating cardiovascular complications accordingly (43). We could conclude from these studies that ANGPTL4 could affect cardiac function mainly by regulating lipid metabolism. Independent of its main function on lipid metabolism regulation, ANGPTL4 can execute other biogical functions as well. Increased glomerular and urinary ANGPTL4 expression has been identified in diabetic rats (44), and hyposialylated ANGPTL4 induces apoptosis of podocytes *via* β1 Integrin/FAK signaling in diabetic nephropathy (45). ANGPTL4 binds neuropilins and cooperates with VEGF to induce diabetic macular edema (46). Furthermore, ANGPTL4 exacerbates pancreatitis by augmenting acinar cell injury through upregulation of C5a (47). In our current study, we focused on the signaling pathway in which integrin was involved, and we could show that ANGPTL4 could promote cardiac apoptosis *via* a FAK/SIRT3/ROS signaling pathway in DbCM.

In addition, ANGPTL4-αvβ3 interaction mediates ANGPTL4 vasoprotective effects (48). However, another study showed that cANGPTL4 activates integrin αvβ1-mediated Rac1/PAK signaling to weaken cell–cell contacts (49). cANGPTL4 interacts with integrins (αvβ1 and αvβ5) and confers anoikis resistance to tumors (22). cANGPTL4 was also reported to modulate keratinocyte migration through interaction with integrins β1 and β5, but not integrin β3 (50). These data indicated a possible cell- and context-dependent interaction of ANGPTL4 and integrins. In our current study, we could only show that FAK, the key effector of integrins, was regulated by ANGPTL4, whereas the specific integrin that was involved in this context and how ANGPTL4 convey its downstream signaling *via* integrin remains unsolved. Thus, we will further uncover the detailed mechanism by which ANGPTL4 interact with specific integrin(s) on cardiomyocyte under DbCM condition in the future study.

Downstream of integrin, we found that SIRT3 could regulate oxidative stress and apoptosis *via* deacetylation of SOD2 in the STZ-induced diabetic hearts. SIRT3 has been shown to increase the expression of Parkin by deacetylating FOXO3A, which induced mitophagy in the heart and promoted cardiomyocyte survival in diabetic rats (51). Sundaresan and colleagues (52) reported that SIRT3 was decreased, accompanied by increased percentage of apoptotic cells in STZ-induced diabetes mouse model. Cardiac function and myocardial properties were significantly worse in SIRT3 knock out mice than wild-type control mice treated with STZ, along with more depressed autophagy. These findings further support our current finding; however, whether autophagy or mitophagy play a pivotal role in our model remains unknown. Future studies with genetic manipulated animal models are required to address this question.

Some limitations should be acknowledged in our study. First, the hub genes were validated only in the animal model, not in human biological samples. Second, this analysis was based on the whole left ventricle heart tissues gene expression profile from GEO. Single-cell transcriptome analyses could give us a much more accurate understanding of the pathophysiology of DbCM. Third, overexpression and knockdown of ANGPTL4 were not performed *in vivo*, which led to insufficient evidence. In summary, through WGCNA, we uncovered ANGPTL4 as a key hub gene in DbCM. Our data suggest that ANGPTL4 can be a potential biomarker and therapeutic target for DbCM.

## Data Availability Statement

The original contributions presented in the study are included in the article/**Supplementary Material**. Further inquiries can be directed to the corresponding authors.

## Ethics Statement

The animal study was reviewed and approved by the Institutional Animal Care and Use Committee of Tongji Medical College, Huazhong University of Science and Technology.

## Author Contributions

LD, HZ, and HW designed the study. LD conducted the bioinformatic analysis. YX and MW established the diabetic mouse model. LD and YX conducted the *ex vivo* and *in vitro* analysis, wrote the first draft of the manuscript, performed the interpretation of the results, and wrote the final version of the article in collaboration, which was critically revised by WZ, XZ, MW, HJ, XL, ZH, and HW. All authors agreed to be accountable for all aspects of the work. All authors contributed to the article and approved the submitted version.

## Funding

The project was supported by the National Natural Science Foundation of China (No. 81600301, No. 81873523, and No. 82070490) to HW and HZ.

## Conflict of Interest

The authors declare that the research was conducted in the absence of any commercial or financial relationships that could be construed as a potential conflict of interest.

## Publisher’s Note

All claims expressed in this article are solely those of the authors and do not necessarily represent those of their affiliated organizations, or those of the publisher, the editors and the reviewers. Any product that may be evaluated in this article, or claim that may be made by its manufacturer, is not guaranteed or endorsed by the publisher.

## Supplementary Material

Supplementary Figure S1 **(A)** Samples clustering to detect outliers (GSE 5606). The clustering was based on the expression data from Normal (n=7) and Diabetic animals LV heart tissue (n=7) at 16 weeks. **(B)** Heatmap depicts the Topological Overlap Matrix (TOM) of genes selected for weighted co-expression network analysis. **(C)** Protein-protein interaction network analysis of all genes in the yellow module. **(D)** Protein-protein interaction network analysis of all genes in the green module. **(E)** Volcano plot of genes detected in diabetic cardiomyopathy. Red means upregulated DEGs; green means downregulated DEGs; gray means no difference. DEG, differentially expressed gene.

Supplementary Figure S2 Twenty-four-hour urine volume at 26 weeks after STZ injection from control and diabetic cardiomyopathy mice (n=6 each). ***p* < 0.01 vs CON group (student *t*-test).

Supplementary Figure S3 Respresentive immunofluorescence staining and quantification of ANGPTL4 in AC16 cardiomyocytes after treatment with HG (30 mM) for 48 h. Scale bar, 50 μm. **p* < 0.05, ***p* < 0.01 *vs* CON group (student *t*-test).
