# Supplementary figures and images for: Weighted Gene Co-Expression Network Analysis Identifies ANGPTL4 as a Key Regulator in Diabetic Cardiomyopathy via FAK/SIRT3/ROS Pathway in Cardiomyocyte
Source: Front Endocrinol (Lausanne). 2021 Sep 20;12:705154. doi: 10.3389/fendo.2021.705154 (PMC8488438; doi:10.3389/fendo.2021.705154)

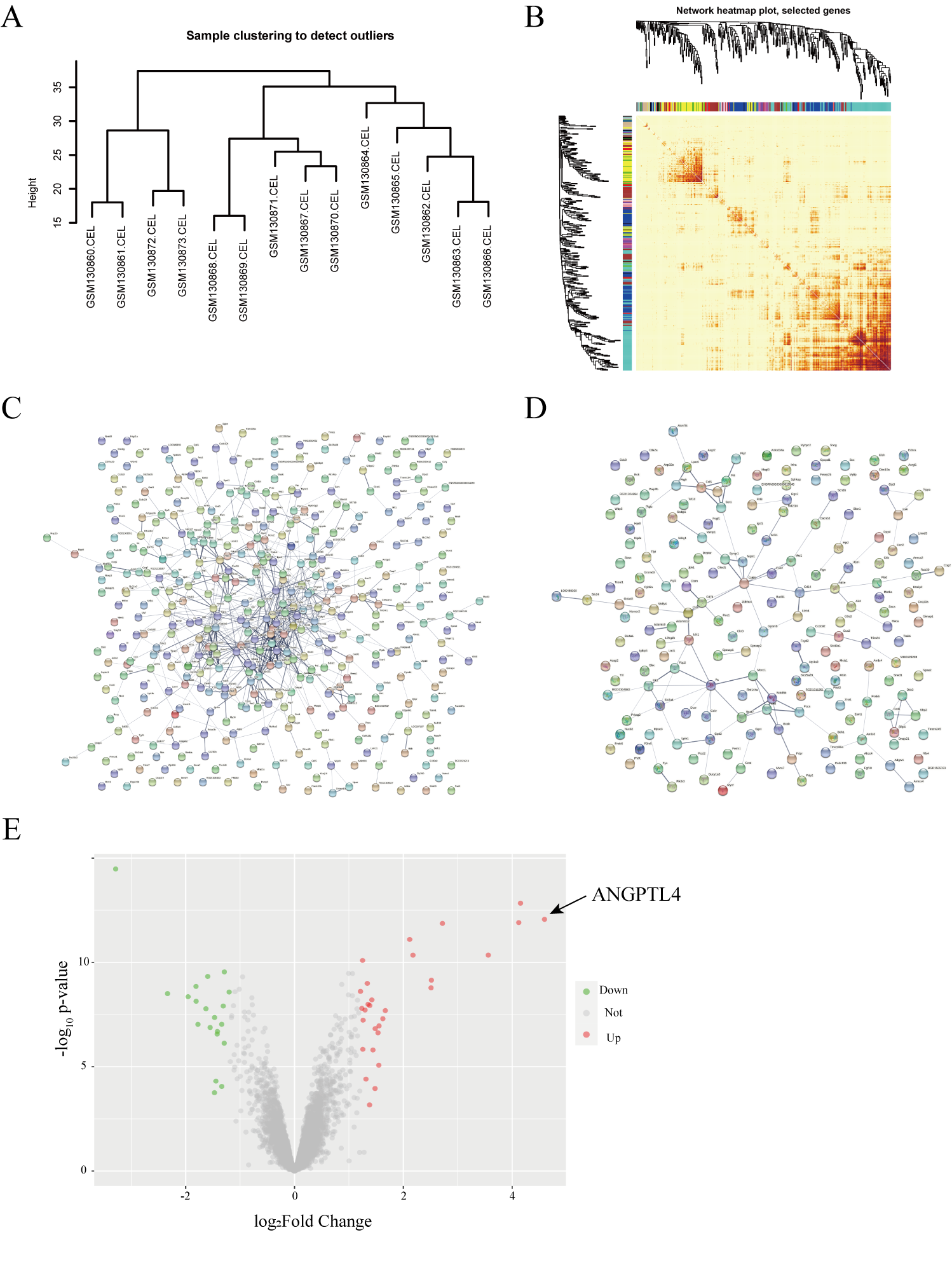

Supplement: Supplementary Figure S1 — (A) Samples clustering to detect outliers (GSE 5606). The clustering was based on the expression data from Normal (n=7) and Diabetic animals LV heart tissue (n=7) at 16 weeks. (B) Heatmap depicts the Topological Overlap Matrix (TOM) of genes selected for weighted co-expression network analysis. (C) Protein-protein interaction network analysis of all genes in the yellow module. (D) Protein-protein interaction network analysis of all genes in the green module. (E) Volcano plot of genes detected in diabetic cardiomyopathy. Red means upregulated DEGs; green means downregulated DEGs; gray means no difference. DEG, differentially expressed gene. [file Image_1.tif]

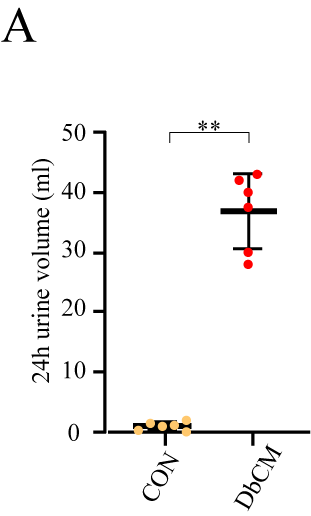

Supplement: Supplementary Figure S2 — Twenty-four-hour urine volume at 26 weeks after STZ injection from control and diabetic cardiomyopathy mice (n=6 each). **p < 0.01 vs CON group (student t-test). [file Image_2.tif]

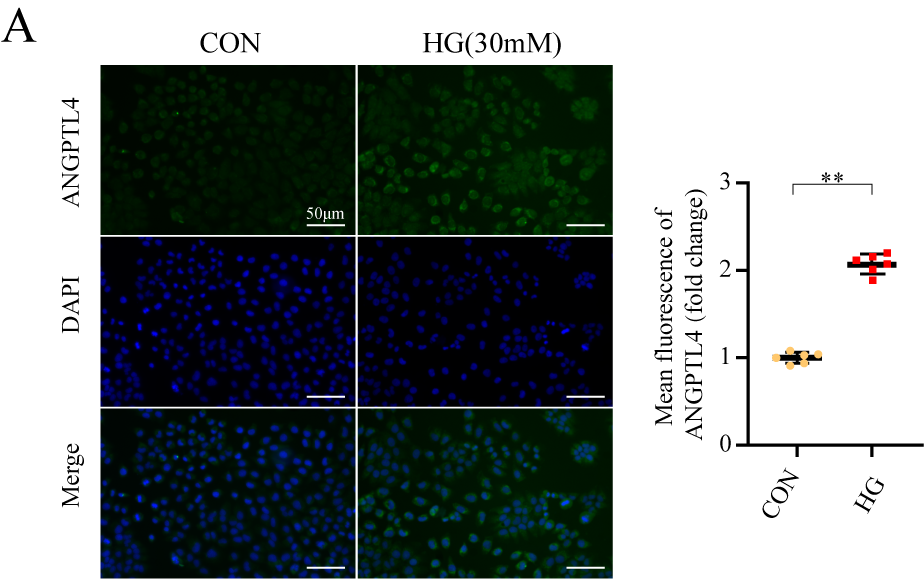

Supplement: Supplementary Figure S3 — Respresentive immunofluorescence staining and quantification of ANGPTL4 in AC16 cardiomyocytes after treatment with HG (30 mM) for 48 h. Scale bar, 50 μm. *p < 0.05, **p < 0.01 vs CON group (student t-test). [file Image_3.tif]
